# *Chunyun* versus lockdown

**DOI:** 10.1093/nsr/nwab178

**Published:** 2021-10-02

**Authors:** Sune Lehmann

**Affiliations:** DTU Compute, Technical University of Denmark, Denmark; Center for Social Data Science, University of Copenhagen, Denmark

Across the world, the news of COVID-19 was followed by ‘lockdowns’ that limited societal activity and mobility to a greater or lesser degree. In China, however, the situation was unique because the travel lockdown occurred in the middle of the year's most hectic travel season occurring around Chinese Lunar New Year (*chunyun*).

It is worth spending a moment to appreciate how massive *chunyun* is as an event. *Chunyun*, or ‘Spring Transport’ in Chinese, is literally the world's largest annual human migration [1], in 2016 estimated to result in almost 3 billion journeys [2]. It starts around 15 days before New Year's Day and lasts for about 40 days.

**Figure 1.**
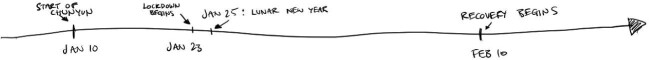
A timeline of events. *Chunyun* starts on 10 January, with the lunar new year occurring on 25 January. A massive lockdown occurred on 23 January 2020, during the height of travel season.

In China, the first COVID-19 lockdown occurred right in the middle of this period of intense travel (see Fig. 1), and the paper ‘Mobility in China, 2020: a tale of four phases’ by Suo-yi Tan and co-authors [3] lays out the remarkable changes in mobility that were associated with the clash of the two opposing events of lockdown and widespread displacement of the largest population on the planet.

To do so, the authors draw on an impressive dataset of 318 million mobile phone users, whose aggregated and anonymized mobility was used to understand how mobility flows were shaped by these unprecedented events—in a way that caused existing models for human mobility to become less predictive.

As revealed by Tan *et al.*, a key to understanding mobility in this intense situation is the Chinese system of prefectures, which has been divided into tiers with the highest tier labeled ‘super-tier’ and subsequent tiers labeled Tiers 1–5. The tier-labels describe each prefecture's relative level of development, with the higher-tiers being more developed.

What happens during a normal *chunyun* is that we observe an enormous migration of individuals from high-tier prefectures flowing to lower-tier prefectures, often traversing very long distances across the country. These travelers are typically workers from less developed areas working in highly developed prefectures, students, etc., traveling home to visit family. This pattern is confirmed by Tan *et al*.

In 2020, however, the normal travel pattern was disrupted by nationwide travel restrictions. These restrictions occurred essentially at the time of the largest number of displaced individuals. Thus China remained in the non-normal state typically associated with ‘peak *chunyun*’ for longer than normal. Only on 10 February were travel restrictions lifted and people could return, with flows normalizing around 29 February. See Fig. 1 for a timeline.

The authors show that we currently lack a well understood model to account for the mobility patterns occurring as *chunyun*, epidemic and lockdown intertwined [3]. It is because of this situation that the standard models of mobility [4,5] cease to be good descriptors of events—especially for short distances, and the model developed by Tan and coauthors is able to shed new light. As one would expect, across the entire time-span, the outgoing trips were matched with return trips. People ended up where they started.

But what the authors show is that, in fact, the backflow was quite different from the outflow. Millions of individuals were able to return *before* the recovery started (the authors name this the ‘backflow effect’). This backflow effect does not imply that most individuals returned before the recovery started. In fact, the authors show that the travel restrictions delayed more than 72.89 million people returning by the end of *chunyun*, mainly for work and education purposes. Instead, the early backflow typically consisted of workers returning to their place of work, traveling between low-tier prefectures, and especially over short distances, while the high-tier prefectures experienced the majority of the delays. Further, using community detection [6] in a novel way, the authors found that the typical geographical communities of flow were disrupted, splintering into smaller regions, once again emphasizing the more regional emphasis during the travel restrictions.

The work by Tan *et al*. [3] is an important empirical documentation of the complex population flows that occur during travel restrictions, especially in a situation of massive displacement during the onset of those restrictions. The authors’ findings, which document and model how society began to slowly return to normal through an unusual increase of short trips by workers returning before the official recovery period, will be valuable to policy makers and epidemic modelers alike.

***Conflict of interest statement.*** None declared.
